# How Overweight and Obesity Relate to the Development of Functional Limitations among Filipino Women

**DOI:** 10.3390/geriatrics3040063

**Published:** 2018-09-27

**Authors:** Linda S. Adair, Paulita Duazo, Judith B. Borja

**Affiliations:** 1 Department of Nutrition, Gillings School of Global Public Health, University of North Carolina at Chapel Hill, Chapel Hill, NC 27599, USA; 2 USC-Office of Population Studies Foundation, Inc., University of San Carlos, Nasipit, Talamban, Cebu City 6000, The Philippines; litlitduazo@yahoo.com; 3 Department of Nutrition and Dietetics, University of San Carlos, Nasipit, Talamban, Cebu City 6000, The Philippines

**Keywords:** obesity, functional limitations, activities of daily living, instrumental activities of daily living, mobility limitations, grip strength, timed up-and-go, Philippines

## Abstract

As life expectancy and obesity increase in low and middle-income countries, the relationship of weight status to functional outcomes in older adults in these settings requires attention. We examined how overweight (BMI > 25 kg/m^2^), obesity (BMI > 30 kg/m^2^), and high waist circumference (WC > 80 cm) related to grip strength, timed up-and-go, and development of limitations in mobility, activities of daily living (ADL), and instrumental activities of daily living (IADL) among Filipino women. We analyzed data from seven rounds of the Cebu Longitudinal Health and Nutrition Survey (1994, *n* = 2279 to 2015, *n* = 1568, age 49–78 years) to examine how women’s reports of functional limitations related to their prior WC, and how their grip strength and timed up-and-go related to concurrently measured overweight and obesity, adjusted for age, socioeconomic status, and urbanicity. High WC was associated with higher odds of subsequent mobility and IADL limitations. Chronic disease morbidity (sum of self-reported arthritis, high blood pressure, heart disease, diabetes, and cancer) fully mediated the association of high WC with ADL and IADL limitations, but not physical/mobility limitations. Longer up-and-go times, and higher grip strength were related to overweight and obesity. Results emphasize the need for obesity prevention to reduce chronic diseases and maintain good functional status as women age.

## 1. Introduction

Obesity has rapidly increased, particularly among lower socioeconomic status (SES) groups in many low and middle-income countries [1]. While a substantial amount of literature relates obesity to cardiometabolic disease risk in low and middle-income countries, relatively little attention has been paid to how obesity relates to functional outcomes and well-being in these settings. For example, in several systematic reviews that identified numerous prospective studies relating obesity to physical disability, all but one of the cited studies were from high income countries [2,3].

Longitudinal studies in high income settings among older adults have found that overweight and obesity are associated with increased odds of developing disabilities. For example, obesity and high WC increased the odds of six-year disability incidence in Dutch adults aged 55 and older [4] and in the US Atherosclerosis Risk in Communities Cohort, obesity at age 25 was associated with increased odds of functional limitations and ADL and IADL impairments later in life [5]. In the Health ABC study, an 8-fold higher risk of incident mobility limitations after age 70 was reported among individuals who were obese since age 25 [6]. In a systematic review, Vincent et al., it was reported that mobility disability (walking, stair climbing, and chair rise ability) was more prevalent with obesity in adults 60 years old and above, and in particular, when BMI exceeded 35 kg/m^2^ [7]. Rejeski and colleagues in their review [2] also noted non-linear effects of BMI, which emphasize the need for studies to examine BMI or high WC categories.

There are several pathways through which obesity may affect physical functioning [2,3]. Excess body weight may directly affect joints and increase risk of osteoarthritis, or alter movement dynamics, postural control, and pain and thus influence mobility [3,6]. The combination of obesity and muscle weakness also relates to functional limitations [8,9,10,11]. Obesity may also increase risk of disability indirectly through its well-known association with chronic diseases, with well-documented associations of diabetes with disability [10,12,13,14,15].

At the same time, physical/mobility limitations may contribute to a more sedentary lifestyle, which, in turn, may lead to weight gain. The relationship of obesity to functional limitations is therefore difficult to understand without using high quality longitudinal data to address the direction of associations and pathways.

Since 1983, the Cebu Longitudinal Health and Nutrition Survey (CLHNS) has collected detailed data from a community-based cohort of women and an index offspring [16]. The CLHNS includes residents of cities and contiguous peri-urban and rural areas that comprise Metro Cebu, the second largest metropolitan area of the Philippines. Metro Cebu exemplifies current demographic and health trends in Asia, where populations are aggregating into rapidly expanding urban centers, levels of economic growth are high, and cardiovascular and related diseases have become the leading cause of mortality. According to 2016 data, heart disease, stroke, and diabetes were the top three conditions responsible for death and disability in the Philippines [17]. Our past research in the CLHNS sample documented a dramatic 7-fold increase in the prevalence of overweight and obesity (BMI > 25 kg/m^2^) from 1986 to 2002 (from 6.5% to 42% of adult women), and a doubling of hypertension prevalence (SBP ≥ 140 or DBP ≥ 90) from 19% to 38% between 1998 and 2007 [18].

Here, we aimed to investigate how overweight and obesity relate to physical capacity (grip strength and timed up-and-go) and to the development of self-reported physical/mobility limitations, activities of daily living (ADLs), and instrumental activities of daily living (IADLs) using data from repeated surveys in the CLHNS cohort. Since the CLHNS includes women across a wide age range, we also assess how these relationships differ by age and over time. We test whether self-reported chronic disease morbidity (elevated blood pressure, diabetes, arthritis, cancer, and heart disease) may mediate a hypothesized association of obesity with disabilities. Moreover, many studies of functional status focus on the elderly and ignore the early development of functional limitations among younger adults.

## 2. Materials and Methods

### 2.1. Sample

Sample women are participants in the ongoing CLHNS [7], which initially recruited all pregnant women in 33 randomly selected urban and rural communities of Metro Cebu who gave birth between 1983 and 1984 (*n* = 3327). All subjects gave their informed consent prior to joining the study. The study was conducted in accordance with the Declaration of Helsinki, and the protocol was approved by Institutional Review Boards at the University of North Carolina at Chapel Hill (IRB #05-1422 and 11-0064) and the University of San Carlos (no number). Here, we use data from full follow-up surveys conducted in 1994 (mean age 38.5 ± 6.0 years, range 25–58), 1998, 2002, 2005, 2012, and 2015 (mean age 58.7 ± 6.0 years, range 49–78). Table 1 shows the sample size and reasons for exclusion from the possible analysis sample from 1994 to 2015, with reasons for loss to follow-up categorized as death, migration out of the Metro Cebu study area or failure to locate, and refusal. As expected death increased over the years as the sample aged, while refusals increased owing to lack of time or waning commitment to participation over a long period of follow-up. A full report of attrition and its consequences for health outcomes is available elsewhere [19].

Table 2 presents mean values of characteristics of women in 1994, among those subsequently lost to follow-up versus those still in the sample in 2015. The retained sample had lower SES as indicated by lower household hygiene, lower income, and less years of education; lived in less urbanized communities; had slightly lower BMI; were less likely to have already reported a physical/mobility limitation; and were less likely to have reported being in poor health.

### 2.2. Measures and Variables

Physical capacity was measured using grip strength (maximum of three hand-held dynamometer readings) in 2005, 2012, and 2015, and the timed up-and-go task [20] measured in 2012 and 2015.

Functional limitations were determined using questionnaires that asked women if they could complete various tasks (a) independently, (b) independently but with difficulty, (c) with help, or (d) not at all. Consistent with other studies, we define two levels of functional limitations: any difficulty or inability to perform the task [21], and severe limitations as able to do tasks only with help or not at all. Tasks were grouped into three categories: physical/mobility (walking 100 m, walking 1 km, standing for 2 h, carrying a weight of 5 kg, climbing a hill or stairs, and doing household chores), activities of daily living (ADLs include ability to lie down after standing, stand up after lying down, eat, bathe, and dress independently), and instrumental activities of daily living (IADLs include ability to shop, prepare food, care for children, use public transportation, and account for money. Physical/mobility limitations were assessed starting in 1994, while ADL and IADL limitations were assessed starting in 1998.

Weight status was represented by BMI and waist circumference (WC). Weight, height, and WC were measured by trained study staff. Overweight and obesity were defined using WHO-recommended BMI cut-points of 25 and 30 kg/m^2^, respectively, and high WC (>80 cm) was used as an indicator of central obesity [22]. Observations were excluded when a woman was pregnant (*n* = 87 in 1994, 51 in 1998, 30 in 2002, 10 in 2005, 1 in 2012).

For morbidity, women were asked whether they had any of the illnesses included in a list read to them. We focused on self-reported high blood pressure (BP), heart disease, diabetes, arthritis, and cancer, which were asked in all survey years. We created a summary index representing the number of illnesses reported from this list, for a potential score of 0 to 5. We assessed the agreement of self-reported high BP and diabetes with high BP measured in triplicate after a 10-min seated rest using OMRON HEM-7211 devices and defined as systolic ≥ 140 mm Hg or diastolic ≥ 90 or taking an antihypertensive medication; and diabetes represented as glycated hemoglobin (NycoCard Reader II) >6.5% or taking medication to treat diabetes. Self-reports underestimated prevalence of hypertension based on measured blood pressure (32.9% vs. 39.1% in 2012; 46.9% vs. 46.5% in 2015) and diabetes based on HbA1c (9.6% vs. 13.2% in 2012, and 13.0% vs. 14.2% in 2015). Self-reports underestimated prevalence of hypertension based on measured blood pressure (32.9% vs. 39.1% in 2012; 46.9% vs. 46.5% in 2015) and diabetes based on HbA1c (9.6% vs. 13.2% in 2012, and 13.0% vs. 14.2% in 2015).

For other covariates, we identified age categories according to age in the baseline year for each analysis (e.g., 1994 for physical/mobility measures, but 2012 for timed up-and-go). The specific age ranges for the groups in each analysis are shown in the results tables, but correspond to ages of 45–54, 55–59, 60–65, and >65 at the last follow-up survey. Socioeconomic status (SES) was represented by the woman’s education (highest grade attained) and an assets score. The assets score was derived from a tetrachoric factor analysis of binary variables representing the quality of housing materials and ownership of household assets such as television, electric fan, air conditioner, tape recorder, refrigerator, vehicles, phones, or livestock. The first factor had an eigenvalue >10 and high factor loadings on items typically owned in wealthier households, such as appliances and vehicles. We used the coefficients representing the factor structure in 2005 to calculate comparable time varying assets scores for all survey years. Higher values represent greater wealth. The urbanicity index was constructed using data from surveys answered by key informants in every community in which a study participant lived. The index (possible range 0–70 points) is the sum of scores for seven components—population size, population density, communications, transportation, educational facilities, health services, and markets. Full details of how the index was calculated, and of scale metrics have been published [23]. History of menstrual periods and presence of menopausal symptoms were queried at each survey and these data were used to create a binary variable indicating, for each survey, whether the woman was postmenopausal or not.

### 2.3. Analysis Methods

We calculated the prevalence of each type of functional limitation and morbidity, and mean grip strength and timed up-and-go across all years. We estimated the likelihood of incident or recurrent physical/mobility, ADL, or IADL disabilities; that is, cases where the woman reported a limitation in the current survey but not in the prior survey, as a function of lagged anthropometric variables and other covariates. We used random effects longitudinal logistic regression models to account for the correlation of repeated measures in individuals (XTLOGIT in Stata 14) [24]. After preliminary analyses to examine age interactions, appropriate functional forms and contribution of SES variables, the disability models included main effects and interactions of age group with lagged weight status, and lagged menopausal status, urbanicity of residence, education, assets, and dummy variables for each survey year. We compared model fit using Bayesian information criteria for models specified alternately with high WC or overweight and obesity, and found that across all incident disability outcomes, high WC models had better fit, so we focus on WC as a measure of central obesity. Preliminary analysis showed no relationship of menopausal status to functional limitations, likely reflecting its collinearity with age, so this variable was dropped from final models.

We assessed the potential mediating role of prior morbidity in the development of disabilities using structural equation models (GSME in Stata 14) [24], accounting for the covariates listed above, and specified with age, but without age interaction terms. The mediation models explored the associations across all survey years, clustered by woman ID to account for the correlation of repeated measures in individuals.

Since physical performance is likely to be more strongly affected by current versus prior weight status, we explored the contemporaneous association of overweight and obesity with grip strength and timed up-and-go using longitudinal linear regression models (XTREG in Stata 14) [24]. For these continuous outcomes, model fit was best with BMI categories that also accounted for non-linear associations (different relationships with overweight vs. obesity). As with functional limitations, menopausal status was unrelated to timed up-and-go and therefore dropped.

To test whether attrition may bias results, we estimated the probability of being in the sample in each year in probit models, with a large set of 1983 baseline socioeconomic and demographic variables as predictors. We then calculated the inverse probability of participation (IPP) for each year, and included this variable as a confounder in all of our models [25]. IPP was not a significant predictor of any outcomes (all *p*-values > 0.10), and the inclusion of IPP in the model did not change other beta coefficients in the model by more than 0.01 units.

## 3. Results

At the last survey (2015), age ranged from 46–79 years, 37% of participants were 60 or older, and 91% were postmenopausal. The CLHNS sample is predominantly low SES: 32% of women did not complete primary school, and 22% had a high school education or more. Mean height is 150 ± 5 cm. Asset and urbanicity scores increased from 1994 to 2015 and more than 90% of participants were postmenopausal at the last survey.

**Figure 1:**
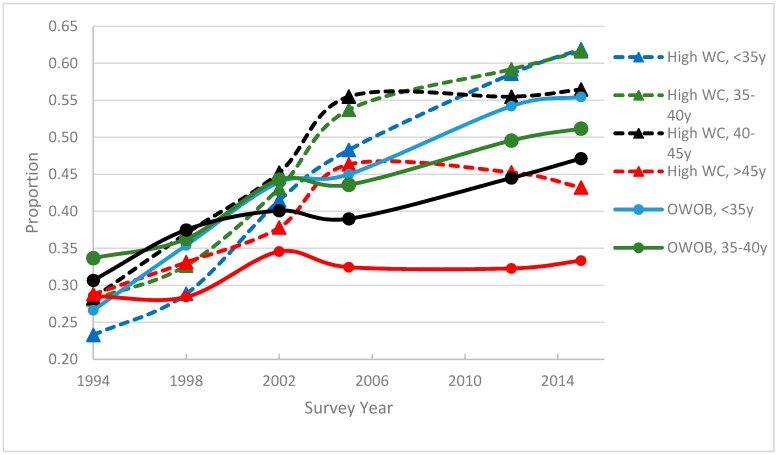
Age and time trends in overweight and obesity (OWOB = BMI > 25 kg/m^2^) and high waist circumference (high WC = WC > 80 cm) among middle to older age Filipino women.

The prevalence of overweight or obesity increased significantly across survey years in all age groups (*p* < 0.01). The prevalence of high WC increased over time in all age groups (Figure 1). The largest increases in prevalence were among women in the youngest age groups. By 2015, more than 60% of women who were <40 years of age in 1994 had a high WC, while 43% of women in the oldest age group had a high WC (*p* < 0.01).

### 3.1. Occurrence of Functional Limitations

Limitations in ability to perform each type of task and of incidence and prevalence of any limitation and any severe functional limitations increased over time (Table 3). Physical/mobility limitations were more prevalent than ADL or IADL limitations, and by 2015, about one-third of women reported at least one physical limitation. Any difficulty climbing stairs or a hill and standing for 2 h were the most commonly reported physical/mobility limitations, reported by about 20% of women in 2015. Severe limitations in ADLs were infrequent across all years. The most common ADL limitation was standing after lying down. Among the IADLs, limitations in ability to care for children, shop, and use public transportation became the most frequent types of IADLs in the later years.

### 3.2. Morbidity

**Figure 2:**
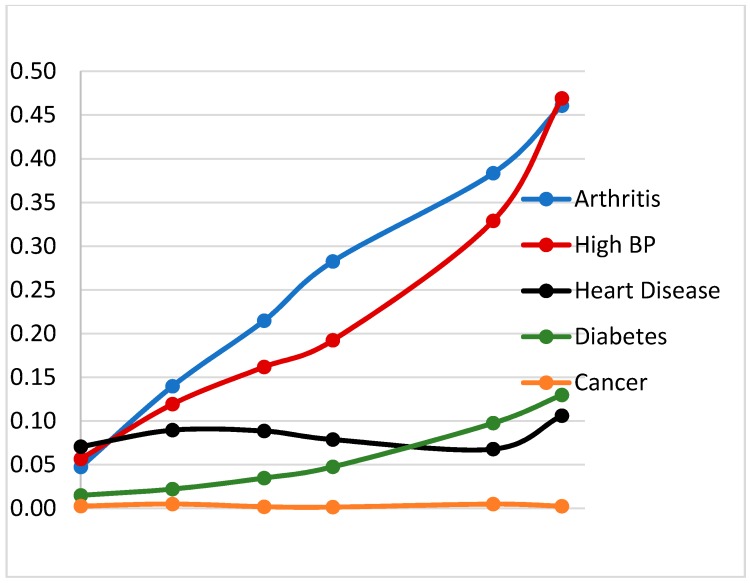
Self-reported morbidity prevalence of Filipino women across all survey years.

Self-reported morbidity increased over time (Figure 2), nearly in parallel across all age groups. By 2015, only 29% of women reported having none of the five illnesses comprising the morbidity index, nearly half of all women reported having arthritis or high blood pressure, and 13% reported having diabetes.

### 3.3. Multivariable Models of Incident Functional Limitations

Odds ratios presented in Table 4 represent the likelihood of any or of any severe incident functional limitation as a function of covariates. Owing to the very low incidence of severe ADL limitations, reliable estimates are not obtainable, so that model is not presented.

The association of lagged high WC with incident severe physical/mobility, any and severe IADLs and any ADLs was modified by age group, as indicated by significant interaction terms. In Table 4, the main effect of lagged high WC (first row of results) represents the odds of experiencing the outcome among women with high WC in the youngest age group. The rows for each interaction term represent the additional effect of lagged high WC in each of the older age groups. For example, using estimates from Table 2, the predicted incidence of a severe physical/mobility limitation in 2015 when this age group was 60–65 years old was two times greater among those with high WC versus those with WC < 80 cm (18.3% vs. 9.4%). Similarly, the predicted incidence of having a severe IADL in that age group was more than three times higher for those with high WC versus those with WC < 80 cm (6.8% vs. 2.0%). Urbanicity was consistently positively associated with all incident functional limitations, but household assets and education were not related to any incident functional limitations.

### 3.4. Mediation Models

**Figure 3:**
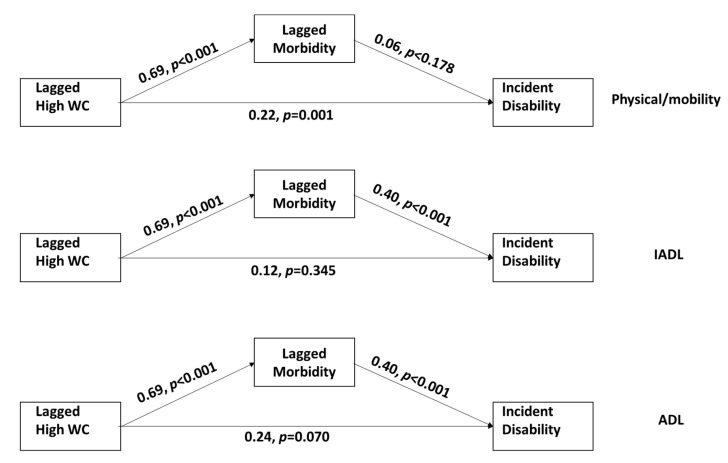
Path models of the association of prior morbidity chronic disease morbidity as a mediator of the association of prior high WC with incident disabilities. Numbers shown on the paths represent beta coefficients.

Path models represent the potential mediating role of morbidity (Figure 3). Numbers on the paths represent beta coefficients from the mediation models. Lagged waist circumference was strongly related to lagged morbidity. Women with high WC, had a mean morbidity score 0.69 units (about 0.8 SD) higher than women with WC < 80 cm. Exponentiating the beta coefficients shown on the paths to incident disability, the observed direct effects represent a 50% increase in the odds of incident IADL and ADL limitations for each one unit increase in the morbidity score (OR = 1.50, 95% CI = 1.32, 1.71 for IADL, and OR = 1.49, 95% CI = 1.31–1.70 for ADL disability). Lagged morbidity was not significantly related to incident physical/mobility disability in the mediation model. The significant direct effect of lagged high WC on physical/mobility limitations translates to an odds ratio of 1.24, 95% CI = 1.10, 1.40.

### 3.5. Grip Strength

Grip strength was higher in younger than older women but declined in all age groups over time (Table 5). The random effects longitudinal regression model showed that grip strength was higher in overweight (2.18 kg (95% CI = 1.27, 3.08)) and obese (4.31 kg (95% CI = 2.91, 5,73)). There were no significant interactions of weight status with age. Other attributes associated with higher grip strength included taller stature, and currently working for pay. Low education and living in a less urban environment were associated with lower grip strength (Table 6).

### 3.6. Timed Up-and-Go

Random effects longitudinal regression models of timed up-and-go in 2012 and 2015 showed longer times in women who were older or overweight or obese (Table 7). Interactions of BMI categories (overweight, obese) with age groups showed that timed up-and-go in women over 60 years of age and with obesity was 4 s (>1 SD) longer than that of normal weight women under 50 years of age. The model also reveals longer times in urban women, those with less education and those not working for pay.

## 4. Discussion

Owing to their relatively young age in 1994, CLHNS participants had a low initial incidence and prevalence of disabilities, but following them for more than 20 years, we were able to observe incident functional limitations associated with age, time, urbanicity, and central obesity. Incident functional limitations increased most sharply after age 60. Physical/mobility limitations were the most prevalent, and among the ADL and IADL tasks, those that required some physical effort (standing, child care, using public transportation) were more prevalent than those requiring less physical effort.

Our repeated measures design and focus on recurrent and incident functional limitations are a key strength of our study. Since having a mobility limitation may decrease physical activity, which in turn contributes to overweight and obesity, our use of lagged measures avoids the potential bias associated with the use of concurrently measured exposures and outcomes.

We found that lagged WC was associated with increased likelihood of any incident physical/mobility limitations across all age groups. Thus, central obesity is an important risk factor for development of mild disability regardless of age. We defined incident disability as reporting a disability at the current survey, but not in the prior one. This allows us to examine recurrent cases as women gain weight and waist circumference over time.

Our mediation analysis showed a significant direct effect of central obesity on incident physical/mobility limitations, even after accounting for the morbidity pathway. This suggests a direct physical effect of excess body weight on ability to perform these types of tasks, even in younger women.

In contrast, lagged high WC was most strongly related to ADL and IADL limitations older women (those >60 at our last survey year). The mediation analysis showed that the influence of central obesity on these limitations is mostly indirect, operating through chronic disease morbidity. However, given the complexity of the ADL and IADL tasks, there may be other pathways related to upper limb function and dexterity not captured in our mediation models.

Central obesity is a well-known risk factor for cardiometabolic diseases [26,27] and our analysis both confirms this and shows the role of such diseases in incident ADL and IADL limitations. In addition, a study in China found waist circumference to be a better predictor than BMI among elderly in Beijing [28]. Our observation that, without accounting for morbidity, central obesity relates to increased likelihood of ADL, IADL, or physical/mobility limitations is consistent with other studies that have explored obesity [29]. In a cross sectional analysis of data from Guatemalan adults >50 years of age, Yount et al. [24] found that overweight was not significantly associated with gross motor disability in women combined after adjustment for physician diagnosed illnesses.

We used two objective measures of physical function: timed up-and-go and grip strength. Women with overweight and obesity, particularly those who were older, had longer timed up-and-go. A study among Indian men and women in a similar age group found that timed up-and-go was not significantly associated with weight (r = 0.009) or BMI (r = 0.058) based on simple correlations [30]. Among Cebu women, the simple correlations (r = 0.019 for weight and r = 0.044 for BMI) were similar to those in the India study [30], but our analysis focused on more extreme values of BMI represented as overweight and obesity.

Among CLHNS participants, grip strength was strongly related to BMI, and women with overweight and obesity having higher grip strength. Based on estimates from bioelectric impedance analysis, the correlation of BMI with muscle mass in out CLHNS participants is 0.71. This may explain the observed positive association. In contrast, a study in South Africa [31] found no association of grip strength with weight status and an Australian study found an inverse association of grip strength with BMI with grip strength in participants between 30 and 70 years of age [32].

Most CLHNS participants are of low SES, and about 75–80% live in highly urban environments. Survey years covered a period of rapid economic change associated with the nutrition transition [33] exemplified by rapid increases in overweight and obesity over the two decades covered in the current analysis. We found that living in a more urban environment was associated with increased incidence of all types of functional limitations. A higher level of urbanization in Cebu is associated with less occupational physical activity, and higher fat, lower quality diets, and these factors contribute to increased BMI and prevalence of overweight and obesity [18]. Women living more traditional lives in rural areas appear to be more protected against these risks.

By 2015, about 60% of CLHNS participants across all ages had a WC > 80 cm, putting them at risk for functional limitations as well as cardiometabolic diseases. Increases in overweight and obesity and high WC were greatest among younger women, while rates leveled off over time in the oldest group. This suggests that the current generation of women who were still 45–60 years of age in 2015 could be facing rapidly increasing morbidity and functional limitations as they age. The lack of association of education, household income, and assets with incident disability suggests that obesity prevention efforts are needed across all SES groups.

Our study has several limitations. First, we used self-reported morbidity data. As this was not a clinical study, we do not have verified diagnoses of arthritis, heart disease, and cancer. Our measured blood pressure and HbA1c data suggest under-reporting of these conditions, likely because they are undiagnosed because of lack of regular screening. Thus, our morbidity score may not have fully captured true disease, and we may have underestimated the effects of morbidity on functional limitations in our mediation analysis. Second, the incidence of severe ADL limitations was quite low, particularly among groups representing high WC and age. Thus, we were not able to estimate that model. Third, as with any long-running cohort study, the CLHNS sample suffered considerable attrition over time. We noted that the retained sample generally had lower SES but lower morbidity and fewer functional limitations than women lost to follow-up. However, our use of inverse probability weighting suggested no attrition bias, meaning that the relationship of overweight, obesity, and high WC to functional limitations was not different in the retained sample and those lost to follow-up.

## 5. Conclusions

In conclusion, in our middle to older aged population in Cebu, we found that central obesity directly affects subsequent physical/mobility, and indirectly affects ADL and IADL limitations through chronic disease morbidity. The trend in increasing central obesity and chronic disease in this population portents a high future burden of disability and emphasize the need for early life prevention of overweight and obesity as a means of minimizing later morbidity and risk of impaired physical functioning or physical/mobility limitations which contribute to poor quality of life.

## Figures and Tables

**Table 1:** CLHNS sample size and reasons for loss to follow-up across survey years. Analysis sample for the current study spans 1994–2015.

Year	In Survey	Died	Migrated or Not Contacted	Refused	Pregnant
1983–1984	3327	0	0	0	
1994	2192	62	951	35	87
1998	1938	86	1200	52	51
2002	2072	110	1039	76	30
2005	2008	136	1087	86	10
2011	1814	251	1138	123	1
2015	1568	314	1242	203	0

**Table 2:** Baseline (1994) characteristics of women lost to follow-up versus retained in the 2015 survey. *p*-values represent chi-square test of proportions for binary variables, and ANOVA for continuous variables, comparing included versus excluded women.

Variable	Lost to Follow-Up (in 1994, not in 2015)		Retained (in 1994 and 2015)		*p*-Value *
	Mean	SD	Mean	SD	
Morbidity score (range 0–3)	0.24	0.54	0.17	0.44	0.002
Hygiene score (range 0–9)	5.41	1.93	5.15	1.88	0.003
Age (years)	38.71	6.35	38.39	5.95	0.250
Education (years)	7.28	3.49	6.75	3.21	<0.001
Log income	5.97	0.88	5.88	0.74	0.012
Urbanicity index (range 8–59)	37.34	12.48	35.10	13.51	<0.001
Height (cm)	150.52	4.96	150.41	5.00	0.622
BMI (kg/m^2^)	23.58	4.19	23.08	3.80	0.004
Reported ≥1 task with difficulty	0.28		0.22		0.001
Reported ≥1 task with severe difficulty	0.06		0.03		<0.001
General health excellent	0.46		0.47		0.679
General health poor	0.02		0.04		<0.001

**Table 3:** Prevalence of any limitations in specific tasks comprising IADLs, ADLs, and physical/mobility limitations, and incidence of any and severe limitations among middle to older age Filipino women across survey years.

**IADLs**										
**Year**	**Handle Accounts**	**Shop**	**Prepare Food**	**Use Public Transit**	**Child Care**		**Any IADL Limitation**	**Any Severe IADL Limitation**	**Any Incident IADL**	**Any Incident Severe IADL**
1998	0.7%	6.3%	1.6%	1.1%	0.5%		6.8%	2.6%		
2002	0.6%	4.9%	1.5%	1.3%	1.2%		5.4%	2.8%	3.2%	1.8%
2005	1.4%	5.2%	1.9%	1.9%	1.0%		6.1%	3.1%	4.7%	2.4%
2012	1.9%	4.4%	1.8%	3.0%	11.8%		5.8%	3.4%	4.3%	2.8%
2015	2.0%	6.5%	2.7%	5.3%	6.0%		8.6%	5.3%	5.8%	3.8%
**ADLs**										
**Year**	**Stand After Sitting**	**Lie Down**	**Shower**	**Eat**	**Dress**	**Use the Toilet**	**Any ADL Limitation**	**Any Severe ADL Limitation**	**Any Incident ADL**	**Any Incident Severe ADL**
1998	5.1%	2.1%	0.3%	0.2%	0.2%	0.4%	5.6%	0.3%		
2002	5.3%	2.2%	0.5%	0.2%	0.4%	0.3%	5.8%	0.4%	4.1%	0.3%
2005	4.7%	2.1%	0.7%	0.4%	0.6%	0.5%	5.4%	0.6%	3.8%	0.5%
2012	3.4%	1.5%	0.9%	0.3%	0.7%	0.8%	3.9%	0.9%	3.0%	0.9%
2015	6.0%	2.7%	1.6%	0.5%	1.5%	1.6%	7.2%	1.3%	5.7%	1.0%
**Physical/Mobility Limitations**										
**Year**	**Walk 100 m**	**Walk 1 km**	**Carry 5 kg Weight**	**Chores**	**Climb Stairs**	**Stand 2 h**	**Any Physical Limitation**	**Any Severe Physical Limitation**	**Any Incident Physical Limitation**	**Any Incident Severe Physical Limitation**
1994	2.9%	5.7%	5.4%	2.7%	13.4%	12.5%	23.6%	4.0%		
1998	2.4%	8.0%	7.3%	6.0%	17.5%	10.2%	24.1%	3.2%	14.6%	2.5%
2002	4.2%	8.2%	5.0%	3.5%	13.3%	10.5%	19.6%	4.0%	10.5%	3.1%
2005	5.6%	8.3%	5.5%	4.6%	10.6%	11.0%	19.1%	6.3%	10.6%	5.2%
2012	6.7%	9.0%	6.3%	4.8%	15.2%	13.7%	22.1%	6.4%	13.9%	5.0%
2015	8.5%	13.0%	7.8%	7.1%	19.8%	20.1%	33.2%	14.8%	19.8%	11.8%

**Table 4:** Association of lagged high waist circumference (WC > 80 cm) with incident functional limitations in Filipino women.

Main Exposures and Covariates	Physical/Mobility		IADL		ADL
	Any OR 95% CI	Severe OR 95% CI	Any OR 95% CI	Severe OR 95% CI	Any OR 95% CI
Lagged high WC	1.26	0.79	1.07	0.88	1.43
	1.00, 1.60	0.52, 1.20	0.66, 1.74	0.45, 1.70	0.85, 2.40
Base (1994) age <35 years	REF	REF	REF	REF	REF
Age 35–40 years	1.14	1.10	1.40	1.45	1.36
	0.91, 1.42	0.76, 1.59	0.89, 2.19	0.81, 2.58	0.82, 2.26
Age 40–45 years	1.49 ***	1.25	1.39	0.92	1.55
	1.18, 1.88	0.83, 1.88	0.84, 2.30	0.44, 1.93	0.89, 2.70
Age 45–50 years	2.18 ***	2.56 ***	3.11 ***	3.72 ***	4.34 ***
	1.73, 2.76	1.77, 3.71	1.99, 4.86	2.14, 6.48	2.69, 7.00
Lagged high WC × age 35–40	1.08	1.79 *	0.77	0.96	0.76
	0.79, 1.49	1.04, 3.08	0.40, 1.48	0.40, 2.28	0.38, 1.54
Lagged high WC × age 40–45	1.07	2.71 ***	1.96 *	4.06 **	1.83
	0.76, 1.50	1.54, 4.79	1.00, 3.83	1.57, 10.46	0.90, 3.72
Lagged high WC × age >45	0.82	1.90 *	1.19	1.05	0.83
	0.57, 1.18	1.09, 3.30	0.63, 2.26	0.45, 2.44	0.43, 1.63
>High school education	1.11	1.00	0.80	0.76	0.94
	0.92, 1.33	0.75, 1.34	0.55, 1.16	0.46, 1.24	0.66, 1.35
Did not complete primary school	0.99	0.99	1.16	1.27	0.87
	0.85, 1.14	0.79, 1.23	0.90, 1.51	0.91, 1.77	0.66, 1.16
Assets score	1.01	1.00	1.01	1.00	0.99
	1.00, 1.03	0.98, 1.02	0.98, 1.03	0.97, 1.03	0.97, 1.02
Urbanicity	1.01 ***	1.02 ***	1.03 ***	1.03 ***	1.03 ***
	1.01, 1.02	1.01, 1.03	1.02, 1.04	1.01, 1.04	1.01, 1.04
1998	REF	REF			
2002	0.63 ***	1.19	REF	REF	REF
	0.52, 0.76	0.82, 1.75			
2005	0.63 ***	2.04 ***	1.47 *	1.34	0.92
	0.52, 0.77	1.44, 2.90	1.06, 2.02	0.87, 2.06	0.67, 1.27
2012	0.80 *	1.78 **	1.23	1.44	0.65 *
	0.66, 0.97	1.24, 2.56	0.88, 1.72	0.94, 2.21	0.45, 0.92
2015	1.23 *	4.63 ***	1.68 **	1.95 **	1.27
	1.02, 1.48	3.32, 6.46	1.21, 2.34	1.28, 2.98	0.93, 1.74
*N*	9379	9379	7396	7396	7396

* *p* < 0.05, ** *p* < 01, *** *p* < 001.

**Table 5:** Maximum of three measures of grip strength of dominant hand (kg), stratified by age group and overweight/obesity status (BMI > 25 kg/m^2^) among middle to older age Filipino women.

Age Group		2005		2012		2015	
		Mean	SD	Mean	SD	Mean	SD
<45	NW	51.7	16.7	46.9	12.4	42.5	10.5
	OWOB	55.6	17.1	50.5 ^a^	12.0	46.1 ^a^	11.4
45–50	NW	50.7	17.3	43.7	12.7	40.9	10.4
	OWOB	50.9	18.3	48.6 ^a^	12.5	44.6 ^a^	10.3
50–55	NW	48.0	17.7	42.9	13.2	38.5	10.5
	OWOB	52.9	17.9	47.6 ^a^	11.8	40.5	10.7
>55	NW	44.6	16.9	40.2	12.7	36.2	9.9
	OWOB	47.2	18.7	43.2	11.7	38.5	10.7

^a^ OWOB>NW within year and age group, ANOVA with Bonferroni correction, all *p*-values < 0.012.

**Table 6:** Association of overweight and obesity with maximum of three measures of grip strength in the dominant hand among middle to older age Filipino women. Results from random effects longitudinal linear regression models.

Main Exposures and Covariates	Coefficient	95% CI		*p*-Value
Normal weight	REF			
Overweight	2.46	1.53	3.39	0.00
Obese	4.99	3.54	6.44	0.00
2005 age <45 years	REF			
45–50 years	−2.33	−3.56	−1.11	0.000
50–55 years	−3.87	−5.27	−2.48	0.000
>55 years	−7.05	−8.63	−5.48	0.000
Assets score	0.04	−0.05	0.12	0.402
>high school education	−0.09	−1.71	1.53	0.918
<primary education	−1.40	−2.56	−0.25	0.017
Urbanicity index	−0.09	−0.13	−0.05	0.000
Currently working for pay	1.04	0.18	1.90	0.017
2005	REF			
2012	−4.21	−4.98	−3.44	0.000
2015	−8.62	−9.43	−7.82	0.000

**Table 7:** Associations of overweight and obesity with timed up-and-go among middle to older age Filipino women. Results from random effects longitudinal linear regression models.

Main Exposures and Covariates	Coefficient	95% CI		*p*-Value
Normal weight				
Overweight (OW)	0.16	0.47	−0.28	0.47
Obese	0.52	0.11	−0.11	0.11
2012 age <50 years				
5055 years	0.17	0.48	−0.29	0.48
55–60 years	0.51	0.04	0.03	0.04
60+ years	1.78	0.00	1.30	0.00
Interaction: age category with weight status				
OW × 50–55 years	−0.08	0.77	−0.65	0.77
OW × 50–55 years	0.28	0.35	−0.31	0.35
OW × 60+ years	−0.23	0.46	−0.86	0.46
Obese × 50–55 years	−0.02	0.97	−0.85	0.97
Obese × 50–55 years	−0.09	0.84	−0.98	0.84
Obese × 60+ years	1.68	0.00	0.61	0.00
Assets score	0.00	0.85	−0.02	0.85
Education (years)	−0.14	0.00	−0.18	0.00
Urbanicity index	0.01	0.05	0.00	0.05
Currently working for pay	−0.27	0.00	−0.43	0.00
Year				
2012	REF			
2015	−0.17	0.00	−0.26	0.00
